# *Streptococcus pneumoniae* Endopeptidase O Promotes the Clearance of *Staphylococcus aureus* and *Streptococcus pneumoniae* via SH2 Domain-Containing Inositol Phosphatase 1-Mediated Complement Receptor 3 Upregulation

**DOI:** 10.3389/fcimb.2020.00358

**Published:** 2020-07-17

**Authors:** Sijie Li, Hong Zhang, Jiangming Xiao, Taixian Yuan, Zhaoche Shu, Yajun Min, Wenchun Xu, Yibing Yin, Xuemei Zhang

**Affiliations:** ^1^Department of Laboratory Medicine, Key Laboratory of Diagnostic Medicine (Ministry of Education), Chongqing Medical University, Chongqing, China; ^2^Department of Laboratory Medicine, The Affiliated Hospital of North Sichuan Medical College, Nanchong, China; ^3^Department of Laboratory Medicine, North Sichuan Medical College, Nanchong, China; ^4^Translational Medicine Research Center, North Sichuan Medical College, Nanchong, China

**Keywords:** PepO, SHIP1, CR3, *S. aureus*, *S. pneumoniae*

## Abstract

Increasing evidences demonstrate that microorganism and their products protect against bacterial and viral pathogens through various mechanisms including immunomodulation. *Streptococcus pneumoniae* endopeptidase O (PepO), a pneumococcal virulence protein, has been proven to enhance the phagocytosis of *Staphylococcus aureus* and *Streptococcus pneumoniae* by macrophages in our previous study, where we detected the down regulation of SH2 domain-containing inositol phosphatase 1 (SHIP1) and the up regulation of complement receptor 3 (CR3) in PepO-stimulated macrophages. In the present study, using SHIP1 over-expression plasmid and CR3 siRNA, we proved that the down regulation of SHIP1 and the up regulation of CR3 mediate the enhanced phagocytosis of *S. aureus* and *S. pneumoniae* by PepO-stimulated macrophages. The down regulation of SHIP1 also mediates the up regulation of CR3. To further determine whether PepO protects against respiratory pathogens, we constructed a mouse model with intranasal infection of *S. aureus* or *S. pneumoniae* and found that PepO significantly promoted their clearance. The down regulation of SHIP1 and the up regulation of CR3 also play a role in this process. This study provides a new preventive and therapeutic option for respiratory infectious diseases and lays the theoretical basis for the development of PepO as an immunomodulation agent.

## Introduction

Infectious diseases account for 6 in 10 threats to global health in 2019 according to the world health organization (WHO), among which antimicrobial resistance has aroused increasing attention. Antibiotic therapy has rescued millions of lives and markedly reduced morbidity and mortality worldwide. Paradoxically, antibiotic therapy simultaneously increases susceptibility to a range of infection and colonization of antimicrobial-resistant pathogens (Buffie and Pamer, 2013; Ng et al., 2013; Theriot et al., 2014; Schubert et al., 2015; Langdon et al., 2016; Fjalstad et al., 2018). To combat antimicrobial-resistant pathogens, many approaches including limits on antibiotic use and development of more effective antibiotics have been implemented. However, the antimicrobial resistance is still growing. There is an urgent need to develop new and more effective ways to combat antimicrobial-resistant pathogens.

An exciting discovery is that administration of protective commensal bacterial species shows potential to reduce antimicrobial-resistant infections (Pamer, 2016; Thiemann et al., 2017; Keith and Pamer, 2019). Several studies have suggested that reestablishment with normal intestinal microbiota reduces colonization by vancomycin-resistant enterococci (VRE), antimicrobial-resistant *Klebsiella pneumoniae* and *Escherichia coli* (Ubeda et al., 2013; Singh et al., 2014; Caballero et al., 2015; Stripling et al., 2015). Many studies have shown that administration of several commensal bacterial species protects against *Clostridium difficile* infection (van Nood et al., 2013; Buffie et al., 2015; Lewis and Pamer, 2017; Deng et al., 2019). However, there are many concerns about developing the live microorganism into preventive or therapeutic agents for their potential pathogenicity, possibility of acquiring antibiotic resistance, and difficult guarantee of purity, uniformity, and effectiveness (Pamer, 2016; Zitvogel et al., 2017). Identifying a single component owing the beneficial effects of live microorganism may be the solution to the above problems (Zitvogel et al., 2017). Several studies have proven that some microbial products protect against viral and bacterial pathogens through various mechanisms including immunomodulation (Steed et al., 2017; Webster et al., 2017; Jacobson et al., 2018).

*Streptococcus pneumoniae* endopeptidase O (PepO) is a ubiquitously expressed pneumococcal virulence protein (Agarwal et al., 2013). Our previous work has proven that PepO enhances the phagocytic function of macrophages in a miR-155 dependent manner (Yao et al., 2017), and macrophages play an important role in the clearance of respiratory pathogens (Lovewell et al., 2014; Byrne et al., 2015; Eichinger et al., 2015; Lemon et al., 2015), indicating that PepO may protect against respiratory pathogens partially through immunomodulation. Even so, the exact molecular mechanisms involved in this process are still unclear. In the previous study, we detected the down regulation of SH2 domain-containing inositol phosphatase 1 (SHIP1) in PepO-stimulated macrophages and proved that SHIP1 down regulation was targeted by miR-155. Several studies have shown that SHIP1 negatively regulates the phagocytic function of macrophages via various mechanisms including inhibiting the release of proinflammatory cytokines and degrading phosphatidylinositol-3,4,5,-trisphosphate at the phagocytic cup (Horan et al., 2007; Cremer et al., 2009). However, whether SHIP1 down regulation is correlated with the phagocytosis by PepO-stimulated macrophages and the related mechanisms remain to be proven.

SHIP1 has been proven to inhibit phagocytic activity mediated by complement receptor 3 (CR3) (Horan et al., 2007). However, we detected the increased expression of CR3 in PepO-stimulated macrophages. Therefore, we speculated that SHIP1 may participate in the regulation of phagocytosis by PepO-stimulated macrophages via modulating CR3 expression level. To test this speculation, we transfected macrophages with pHBLV-CMV-SHIP1 plasmid for over-expression of SHIP1 or with CR3 siRNA for knock down of CR3 and then explored the effect of PepO on these cells' phagocytic function. In the present study, we showed that the enhanced phagocytosis of *S. aureus* and *S. pneumoniae* by PepO-stimulated macrophages was mediated by the down regulation of SHIP1 and the up regulation of CR3. SHIP1 down regulation also mediated CR3 up regulation. To determine whether PepO protects against respiratory pathogens, we constructed a mouse model with intranasal infection of *S. aureus* or *S. pneumoniae* and found that PepO significantly promoted their clearance. The enhanced clearance of *S. aureus* and *S. pneumoniae* also correlated with the down regulation of SHIP1 and the up regulation of CR3. This study provides a new preventive and therapeutic option for respiratory infectious diseases and lays the theoretical basis for the development of PepO as an immunomodulation agent.

## Materials and Methods

### Mice

Specific-pathogen-free male and female, 6–8 weeks old C57BL/6 mice were purchased from Beijing HFK Bioscience Co., Ltd. (Beijing, China) and maintained at Chongqing Medical University. All mice were maintained with sterile water and mouse chow *ad libitum* under barrier conditions. All experimental procedures were approved by the Ethics Committee of Chongqing Medical University.

### Bacterial Strains

D39, a standard strain of *S. pneumoniae*, was purchased from the American Type Culture Collection (ATCC). A standard strain of *S. aureus* numbered ATCC 29213 was obtained from the Children's Hospital of Chongqing Medical University. The strains were seeded on the Columbia sheep blood agar (PANGTONG, Chongqing, China) and cultured in 5% CO_2_ at 37°C overnight. Then the bacteria were suspended in phosphate buffer solution (PBS) with a final OD_600_ value of 0.5.

### Preparation of PepO Protein

Preparation of PepO protein has been described in details previously (Yao et al., 2017). The Ni^2+^-charged column chromatograph used for PepO purification was purchased from GE Healthcare (Buckinghamshire, United Kingdom). Polymyxin B agarose used for lipopolysaccharide (LPS) removal was purchased from Genscript Corp. (New Jersey, USA). The PepO preparation contained no detectable LPS when it was detected by the Limulus Amoebocyte Lyase assay, and the concentrations of rPepO preparation were determined by BCA assay.

### Cell Culture

Four days after intraperitoneal injection of 1 ml paroline, 3–5 male C57BL/6 mice were sacrificed for peritoneal exudate macrophages (PEMs) isolation. Male mice were used to collect more cells. The cells were harvested by peritoneal lavage with 13 ml sterile PBS containing 5 mM EDTA. After centrifugation and washing with Dulbecco's modified Eagle's medium (DMEM) (HyClone, Barrington, IL, USA), the total viable cell numbers were determined with the use of a Neubauer chamber and 5% trypan blue solution. Then the cells were seeded on 6-well cell culture plate (8 × 10^6^/well) or 24-well cell culture plate (5 × 10^5^/well) and cultured in 5% CO_2_ at 37°C for 1 h. After removal of suspension cells, the remaining adherent cells were cultured in DMEM supplemented with 10% heat-inactivated (56°C, 30 min) fetal bovine serum (FBS) (Biological Industries, Kibbutz Beit Haemek, Israel) and 1% penicillin-streptomycin (HyClone, Barrington, IL, USA) and treated as described below. Flow cytometry analysis was used to determine the purity of cultured cells. The results showed that the percentage of macrophages was above 90% (data not shown), so we did not detect its purity in the latter experiments.

### Cell Transfection Assay

The siRNAs (siSHIP1, siCR3, and scrambled siRNA) were purchased from Chongqing LaiBoSi Biotechnology Co., Ltd. (Chongqing, China). Their sequences are listed in Table 1. Plasmids pHBLV-CMV-SHIP1 and pHBLV-CMV-MCS were purchased from HanHeng Biotechnology Co., Ltd. (Shanghai, China). PEMs were transfected with siRNA or plasmid using Lipofectamine 2000 (Invitrogen) according to the manufacture's instruction. After incubation of siRNA or plasmid DNA with Lipofectamine 2000 in appropriate proportions and fixed volumes for 20 min, the liposome-DNA mixture was added into PEMs cultured in serum-free and antibiotic-free DMEM. Five hours later, the cells were cultured in DMEM supplemented with 10% heat-inactivated FBS and 1% penicillin-streptomycin for another 24 h.

**Table 1:** Primers used in this study.

**Primer name**	**Nucleotide sequence (5^**′**^-3^**′**^)**
SHIP1 forward	ACTTTGCTGGAGTGTCCGT
SHIP1 reverse	TTGGGCAGAATCCTGTAAG
CR3 forward	GAGGCCCCCAGGACTTTAAC
CR3 reverse	CTTCTTGGTGAGCGGGTTCA
si SHIP1	UGCAAGAAGUCACCAGCAU
	AUGCUGGUGACUUCUUGCA
si CR3	GCAUCACCAUGAGUGCCAU
	AUGGCACUCAUGGUGAUGC

### Quantitative PCR (Q-PCR) Analysis

Total RNA was extracted from transfected PEMs with the use of RNAiso Plus reagent (TaKaRa) and reversely transcribed into cDNA with the use of PrimeScript RT reagent Kit (TaKaRa) according to the manufacture's instruction. For quantitative analysis, the mixture of cDNA, primers, and TB Green premix EX TaqII (TaKaRa) was amplified in a Bio-Rad real-time PCR machine at an annealing temperature of 60°C and for an extension time of 10 s. The amplification for GAPDH was used as endogenous reference. After completion of the amplification, the Bio-Rad software was used to analyze the PCR products. The relative ΔΔ CT values were used for determining quantification and the relative expressions of SHIP1 and CR3 in vector plasmid and scrambled siRNA groups were used as controls. The primers used are listed in Table 1.

### Western Blot Analysis

After washing with pre-cold PBS, transfected PEMs were lysed with the mixture of RIPA (BiYunTian, Shanghai, China) and SDS loading buffer. Then the samples were collected, boiled for 10 min, and centrifuged at 12,000 g for 10 min to remove cell debris. An equal volume of protein was separated by SDS-PAGE and transferred to a PVDF membrane (Millipore, Bedford, MA). After blocking with 5% defatted milk at 37°C for 2 h, the membrane was probed with anti-SHIP1 (Millipore, Bedford, MA) or anti-CR3 (Novusbio, CO, USA) monoclonal antibody at 4°C overnight. After washing for 3 times, the membrane was incubated with corresponding horseradish peroxidase-labeled secondary goat anti-mouse or goat anti-rabbit antibodies at 37°C for 1 h followed by 4 washing procedures. The antigen-antibody complexes were detected using a Bio-Rad chemiluminescence detection system, and the protein expression was quantified using Quantity one software.

### Phagocytosis Assay

For quantitative analysis, the transfected PEMs were stimulated with PepO for 24 h and infected with *S. aureus* at a MOI of 1:50 or D39 at a MOI of 1:100 at 37°C for 30 min, a time point when there is no bacterial death. After washing with PBS for 3 times, 100 μl pre-cold double distilled water (ddH_2_O) was added. The samples were collected and placed at 4°C for 15 min to fully burst the PEMs. Following a series of dilution, the bacteria were seeded on the Columbia sheep blood agar and cultured in 5% CO_2_ at 37°C overnight. The bacterial colonies were counted. For qualitative analysis, the transfected PEMs were stimulated with PepO for 24 h and infected with FITC-labeled *S. aureus* at a MOI of 1:50 or FITC-labeled D39 at a MOI of 1:100 at 37°C for 30 min. After washing with PBS for 3 times, PEMs were fixed with 0.4 ml 4% paraformaldehyde for 15 min followed by 3 washing procedures and stained with DAPI for 10 min followed by another 3 washing procedures. The phagocytic bacteria were observed under a Nikon Eclipse 80i microscope equipped with a Nikon Intensilight C-HGFI.

### Flow Cytometry Analysis

PEMs were collected and washed twice with pre-chilled PBS. After blocking with CD16/32 antibody for 20 min, cells were stained with APC-labeled CD11b antibody in the dark for 30 min. After washing with PBS for 2 times, the cells were resuspended with PBS for analysis.

### Intranasal Infection of Mice

Female C57BL/6 mice were used for infection experiments because they are more susceptible to *S. pneumoniae*. They were anesthetized with pentobarbital and held in a supine position with the head down. Thirty microliter PBS containing a single compound (30 μg pHBLV-CMV-SHIP1 or pHBLV-CMV-MCS plasmid, 0.4 nmol siCR3 or scrambled siRNA, 10 μg PepO, 1 × 10^8^ CFUs of *S. aureus* or D39) was dropped slowly into their nares with a micropipette at an interval of 24 h. Plasmids pHBLV-CMV-SHIP1 and pHBLV-CMV-MCS, siCR3, and scrambled siRNA were used for *in vivo* transient transfection (Oh et al., 2001; Darcan-Nicolaisen et al., 2009). Twenty-four or forty-eight hours later, the mice were sacrificed for collection of nasal lavage fluids, lung tissues, and blood. The number of viable organisms were determined by counting bacterial colony-forming units (CFUs) on the Columbia sheep blood agar.

### Statistical Analysis

All analyses were performed with the use of Prism 5 statistical software (La Jolla, CA, USA). The data are shown as mean ± standard deviation (SD). Difference between groups was determined by two-way ANOVA test or student's *t*-test. For all experiments, difference with *P* < 0.05 was considered significant.

## Results

### The Down Regulation of SHIP1 Mediates the Enhanced Phagocytosis of *S. aureus* and *S. pneumoniae* by PepO-Stimulated Macrophages

**Figure 1:**
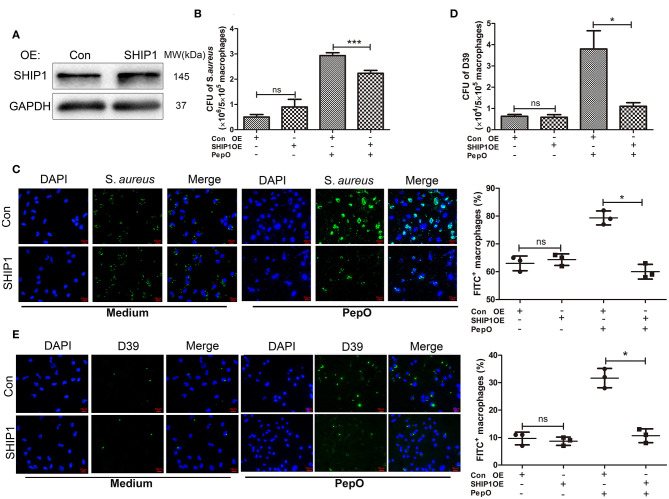
The down regulation of SHIP1 mediates the enhanced phagocytosis of *S. aureus* and *S. pneumoniae* by PepO-stimulated macrophages. **(A)** Immunoblot showed the effective over-expression of SHIP1 in PEMs. **(B,D)** PEMs were transfected with pHBLV-CMV-SHIP1 plasmid or pHBLV-CMV-MCS control plasmid for 24 h followed by PepO stimulation for another 24 h and infection of *S. aureus* **(B)** or D39 **(D)** for 30 min. The number of phagocytosed bacteria was determined. Data are shown as averaged (±SD) bacterial number of three independent repeated experiments. **(C,E)** PEMs were treated as described in **(B)** and infected with FITC-labeled *S. aureus* **(C)** or FITC-labeled D39 **(E)** for 30 min. Representative pictures (original magnification, ×40) from one of the three experiments are shown. The quantification is shown as the averaged (±SD) percent of FITC^+^ macrophages from three independent repeated experiments. Scale bar, 10 μm. Statistical analysis was performed by two-way ANOVA test. ***p < 0.001, *p < 0.05, ns, not significant; OE, over-expression; Con, control.

Our previous study has proven that PepO stimulation leads to the down regulation of SHIP1 in macrophages (Yao et al., 2017). It is still unclear that whether the down regulation of SHIP1 mediates the enhanced phagocytosis of *S. aureus* and *S. pneumoniae* by PepO-stimulated macrophages. To answer this question, we transfected PEMs with pHBLV-CMV-SHIP1 plasmid for over-expression of SHIP1, then the effect of PepO on the phagocytic function of macrophages was determined. Figure 1A shows the effective over-expression of SHIP1 in transfected PEMs without PepO treatment. The number of phagocytosed *S. aureus* and D39 within PepO-treated macrophages was significantly smaller in the SHIP1 over-expression group than in the control group, with more obvious decrease of D39 number (Figures 1B,D). In addition, macrophages which internalized FITC labeled *S. aureus* and D39 were shown in Figures 1C,E. The percentage of FITC positive macrophages was significantly decreased in the SHIP1 over-expression group with PepO treatment compared with the control group, with a similar percentage to the medium group. The quantification was shown in the right panels of Figures 1C,E. Interestingly, Figure 1B shows an increased tendency in the bacterial number of SHIP1 over-expression group in the absence of PepO. We detected CR3 level in macrophages with SHIP1 over-expression and found that it was slightly increased in the absence of PepO (Figure 3B), which to some extent explained this increased tendency. However, in the absence of PepO, CR3 level in macrophages with SHIP1 over-expression did not decrease as expected, which suggests that CR3 expression regulation by SHIP1 should be more complicated. Taken together, these results indicate that the down regulation of SHIP1 mediates the enhanced phagocytosis of *S. aureus* and D39 by PepO-stimulated macrophages.

### The Up Regulation of CR3 Mediates the Enhanced Phagocytosis of *S. aureus* and *S. pneumoniae* by PepO-Stimulated Macrophages

**Figure 2:**
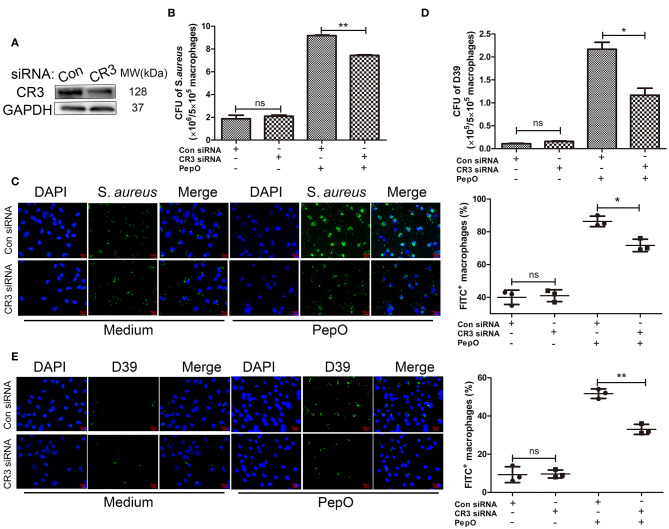
The up regulation of CR3 mediates the enhanced phagocytosis of *S. aureus* and *S. pneumoniae* by PepO-stimulated macrophages. **(A)** Immunoblot showed the effective knockdown of CR3 in PEMs. **(B,D)** PEMs were transfected with CR3 siRNA or scrambled siRNA for 24 h followed by PepO stimulation for another 24 h and infection of *S. aureus* **(B)** or D39 **(D)** for 30 min. The number of phagocytosed bacteria was determined. Data are shown as averaged (±SD) bacterial number of three independent repeated experiments. **(C,E)** PEMs were treated as described in **(B)** and infected with FITC-labeled *S. aureus* **(C)** or FITC-labeled D39 **(E)** for 30 min. Representative pictures (original magnification, ×40) from one of the three experiments are shown. The quantification is shown as averaged (±SD) percent of FITC^+^ macrophages from three independent repeated experiments. Scale bar, 10 μm. Statistical analysis was performed by two-way ANOVA test. **p < 0.01, *p < 0.05, ns, not significant; Con, control.

Our previous study has proven that PepO stimulation leads to the up regulation of CR3 in macrophages. To determine whether the up regulation of CR3 mediates the enhanced phagocytosis of *S. aureus* and *S. pneumoniae* by PepO-stimulated macrophages, we transfected PEMs with CR3 siRNA for knock-down of CR3, then the effect of PepO on the phagocytic function of macrophages was determined. Figure 2A shows the effective knock-down of CR3 in PEMs. As shown in Figures 2B,D, the number of phagocytosed *S. aureus* and D39 within PepO-treated macrophages was significantly smaller in the CR3 siRNA group than in the control group, with more obvious decrease of D39 number. Moreover, the percentage of FITC positive macrophages was significantly decreased in the CR3 siRNA group with PepO treatment, as compared with the control group (Figures 2C,E). The quantification was shown in the right panels of Figures 2C,E. Unexpectedly, we did not see any effect with CR3 knock down in the absence of PepO. A possible reason for this finding maybe that other signaling pathways responsible for phagocytosis compensate for CR3 knock down in macrophages, and that the phagocytosed bacteria in the absence of PepO are relatively less. These results indicate that the up regulation of CR3 mediates the enhanced phagocytosis of *S. aureus* and D39 by PepO-stimulated macrophages.

### The Down Regulation of SHIP1 Mediates the Up Regulation of CR3 in PepO-Stimulated Macrophages

**Figure 3:**
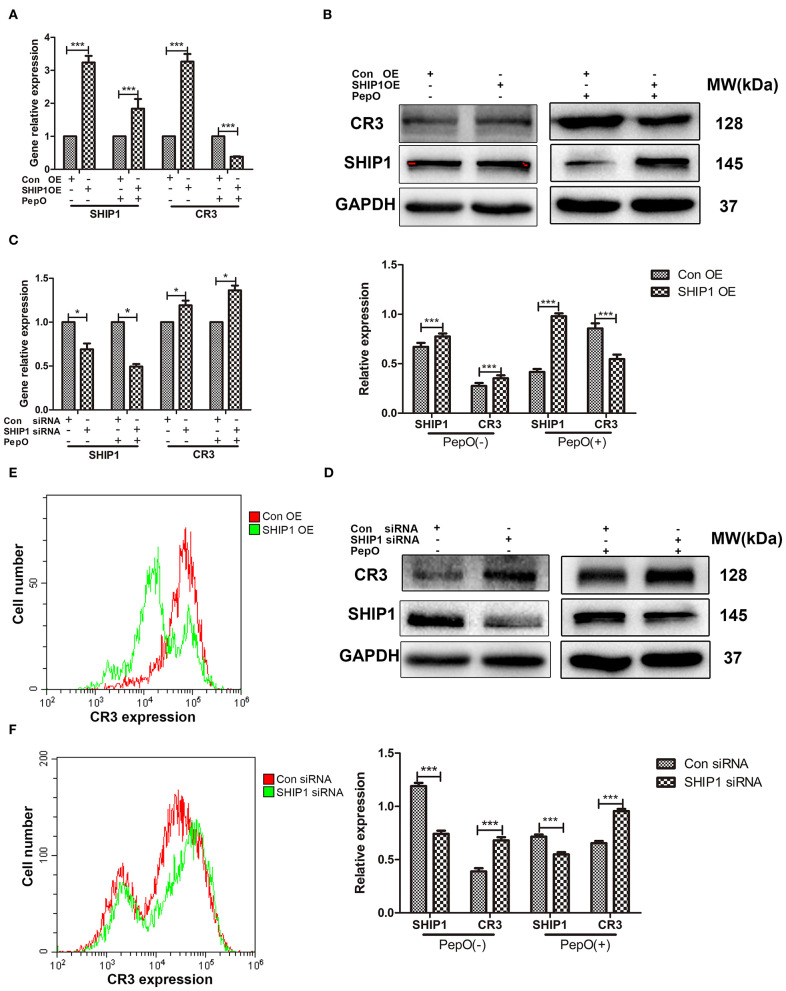
Regulation of CR3 expression by SHIP1 in PepO-stimulated PEMs. **(A–F)** PEMs were transfected with pHBLV-CMV-SHIP1 plasmid, pHBLV-CMV-MCS control plasmid **(A,B,E)**, SHIP1 siRNA, or scrambled siRNA **(C,D,F)** for 24 h followed by PepO or medium stimulation for another 24 h. SHIP1 and CR3 transcripts were determined by Q-PCR analysis, and SHIP1 protein was determined by WB analysis. CR3 protein was determined by WB and flow cytometry analysis. Representative bands from three independent repeated experiments are shown. Data are shown as mean (±SD) of three independent repeated experiments. Statistical analysis was performed by two-way ANOVA test. ***p < 0.001, *p < 0.05, Con, control; OE, over-expression.

Previous studies have shown that SHIP1 negatively regulates the activity of CR3 (Dianne et al., 2001; Horan et al., 2007). It is still unclear that whether the expression of CR3 is regulated by SHIP1 in the current system. To answer this question, PEMs were transfected with pHBLV-CMV-SHIP1 plasmid or SHIP1 siRNA followed by PepO stimulation, then the expression of CR3 was determined. Figures 3A,B shows that both CR3 transcripts and protein were decreased in macrophages with SHIP1 over-expression in the presence of PepO. In contrast, they were increased in macrophages with SHIP1 knock down in the presence of PepO (Figures 3C,D). The quantification of the protein levels of SHIP1 and CR3 was shown in the lower panels of Figures 3B,D. We also used flow cytometry analysis to measure CR3 levels on transfected macrophages with PepO treatment. As shown in Figures 3E,F, CR3 level was decreased in SHIP1 over-expression group while increased in SHIP1 siRNA group compared with the control group, which is consistent with the western blot analysis. These results indicate that SHIP1 negatively regulates the expression of CR3 in PepO-stimulated macrophages, and the down regulation of SHIP1 mediates the up regulation of CR3 in our experiments.

### PepO Promotes the Clearance of *S. aureus* and *S. pneumoniae* in C57BL/6 Mice

**Figure 4:**
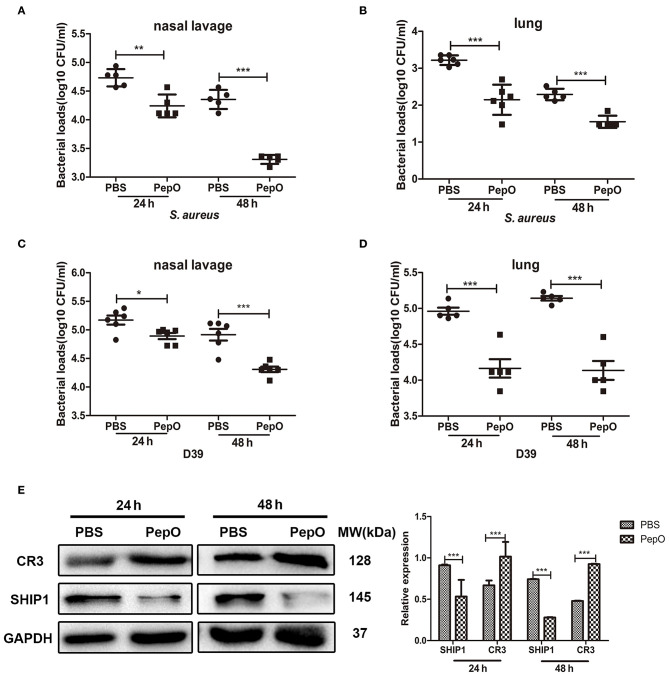
PepO promotes the clearance of *S. aureus* and *S. pneumoniae* in C57BL/6 mice. **(A–D)** C57BL/6 mice were intranasally administrated with PepO or an equivalent volume of PBS for 24 h followed by an intranasal infection of *S. aureus* **(A,B)** or D39 **(C,D)** for 24 or 48 h. The number of viable *S. aureus* or D39 in nasal lavage liquids and lungs was determined. Data are shown as averaged (±SD) bacterial loads of at least five mice per group from one representative experiment of two. **(E)** Immunoblot showed the expression of SHIP1 and CR3 in cells from bronchoalveolar lavage fluids after 24 and 48 h PepO treatment. Representative bands from three independent repeated experiments are shown. Statistical analysis was performed by student's *t*-test. ***p < 0.001, **p < 0.01, *p < 0.05.

Many studies have shown that macrophages play a dominant role in the clearance of respiratory pathogens (Lovewell et al., 2014; Byrne et al., 2015; Eichinger et al., 2015; Lemon et al., 2015). PepO can enhance the phagocytic function of macrophages, indicating that it owes the potential as an immunomodulation agent. Whether it protects against respiratory pathogens remains to be determined. To address this question, female C57BL/6 mice were administrated intranasally with PepO followed by an intranasal infection of *S. aureus* or D39, a standard strain of *S. pneumoniae*. Twenty-four or forty-eight hours later, the mice were sacrificed and their nasal lavage fluids and lungs were harvested for determination of the number of viable *S. aureus* or D39. As shown in Figures 4A–D, the number of viable *S. aureus* or D39 in nasal lavage fluids and lung tissues was significantly smaller in the PepO group than in the PBS group at each time point, with the highest decrease occurring in the lung tissues of PepO group at 48 h. These results indicate that PepO promotes the clearance of *S. aureus* and D39 and alleviates their infection in C57BL/6 mice.

Our above results have shown that the enhanced phagocytosis of *S. aureus* and D39 by PepO-stimulated macrophages depends on the down regulation of SHIP1 and the up regulation of CR3 (Yao et al., 2017). To determine whether the enhanced clearance of *S. aureus* and D39 in PepO-treated mice is associated with SHIP1 and CR3, we detected the expression of SHIP1 and CR3 in cells from bronchoalveolar lavage fluids. Figure 4E shows that the SHIP1 protein was down regulated in the PepO group compared with the PBS group, while the CR3 protein was up regulated in the PepO group compared with the PBS group both after 24 and 48 h PepO treatment. The quantification of SHIP1 and CR3 levels was shown in the right panel of Figure 4E. These results suggest that SHIP1 and CR3 are associated with the enhanced clearance of *S. aureus* and D39 in PepO-treated mice.

### The Down Regulation of SHIP1 Plays a Role in the Enhanced Clearance of *S. aureus* and *S. pneumoniae* in PepO-Treated Mice

**Figure 5:**
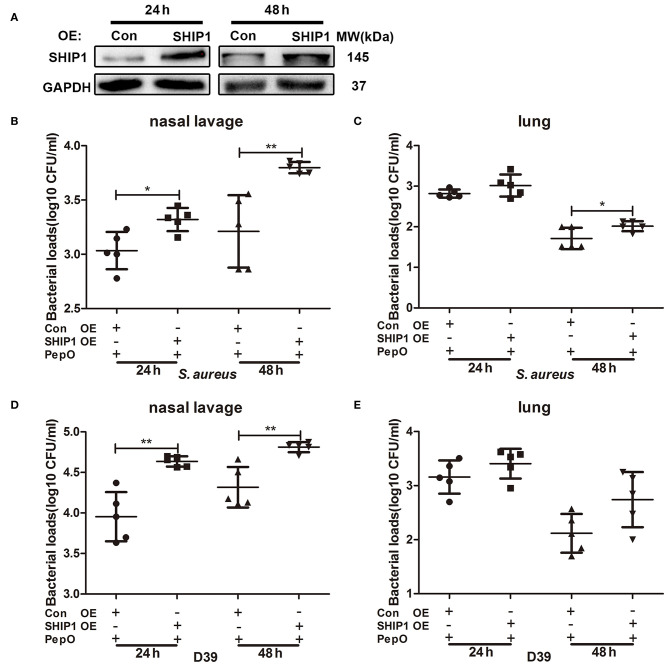
The enhanced clearance of *S. aureus* and *S. pneumoniae* in PepO-treated mice is associated with the down regulation of SHIP1. **(A)** C57BL/6 mice were transiently transfected with pHBLV-CMV-SHIP1 plasmid or pHBLV-CMV-MCS control plasmid via intranasal administration for 24 and 48 h. The effective over-expression of SHIP1 was determined by WB analysis. **(B–E)** After transfected with pHBLV-CMV-SHIP1 plasmid or pHBLV-CMV-MCS control plasmid, mice were intranasally administrated with PepO for another 24 h followed by an intranasal infection of *S. aureus* **(B,C)** or D39 **(D,E)** for 24 and 48 h. The number of viable *S. aureus* or D39 in nasal lavage fluids and lungs were determined. Data are shown as averaged (±SD) bacterial loads of at least five mice per group from one representative experiment of two. Statistical analysis was performed by student's *t*-test. **p < 0.01, *p < 0.05, OE, over-expression; Con, control.

To determine whether the down regulation of SHIP1 is necessary for the enhanced clearance of *S. aureus* and D39 in PepO-treated mice, female C57BL/6 mice were transiently transfected with pHBLV-CMV-SHIP1 plasmid via intranasal administration, then the effect of PepO on the clearance of *S. aureus* and D39 was examined. SHIP1 level in cells from bronchoalveolar lavage fluids was measured by western blot analysis, and the effective over-expression of SHIP1 was shown in Figure 5A. As shown in Figures 5B,D, the bacterial loads in nasal lavage fluids were significantly increased in the SHIP1 over-expression group at each time point with PepO treatment, as compared with the control group. The bacterial loads of *S. aureus* in lung tissues at 48 h were also significantly higher in the SHIP1 over-expression group than in the control group with PepO treatment. Although there was no significant difference in bacterial loads of D39 in lung tissues between the two groups with PepO treatment, the bacterial loads in SHIP1 over-expression group showed an increased tendency compared with the control group (Figures 5C,E). These results indicate that the down regulation of SHIP1 plays a role in the enhanced clearance of *S. aureus* and D39 in PepO-treated mice.

### The Up Regulation of CR3 Plays a Role in the Enhanced Clearance of *S. aureus* and *S. pneumoniae* in PepO-Treated Mice

**Figure 6:**
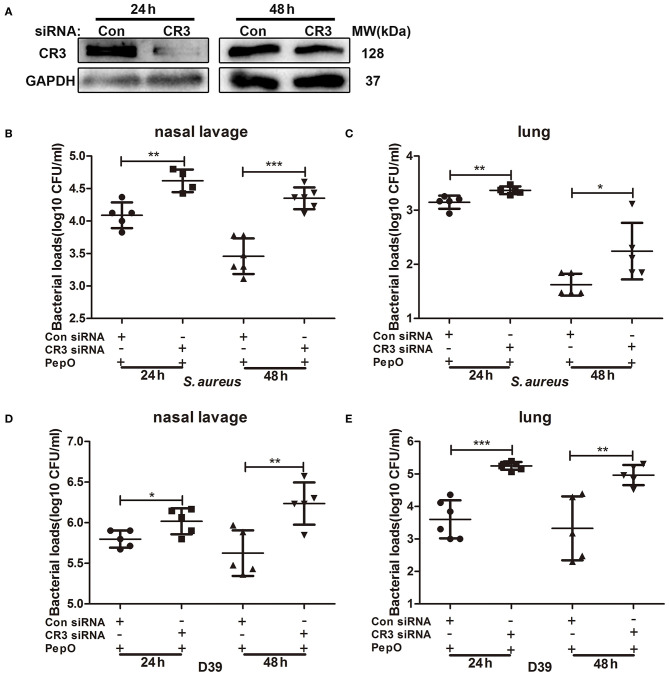
The enhanced clearance of *S. aureus* and *S. pneumoniae* in PepO-treated mice is associated with the up regulation of CR3. **(A)** C57BL/6 mice were transiently transfected with CR3 siRNA or scrambled siRNA via intranasal administration for 24 and 48 h. The effective knock down of CR3 was determined by WB analysis. **(B–E)** After transfected with CR3 siRNA or scrambled siRNA, mice were intranasally administrated with PepO for another 24 h followed by an intranasal infection of *S. aureus* **(B,C)** or D39 **(D,E)** for 24 and 48 h. The number of viable *S. aureus* or D39 in nasal lavage fluids and lungs were determined. Data are shown as averaged (±SD) bacterial loads of at least five mice per group from one representative experiment of two. Statistical analysis was performed by student's *t*-test. ***p < 0.001, **p < 0.01, *p < 0.05, Con, control.

To determine whether the up regulation of CR3 is necessary for the enhanced clearance of *S. aureus* and D39 in PepO-treated mice, female C57BL/6 mice were transiently transfected with CR3 siRNA via intranasal administration, then the effect of PepO on the clearance of *S. aureus* and D39 was examined. CR3 level in cells from bronchoalveolar lavage fluids was measured by western blot analysis, and the effective knock-down of CR3 was shown in Figure 6A. Figures 6B–E shows that the bacterial loads in nasal lavage fluids and lung tissues were significantly higher in the CR3 siRNA group than in the control group at each time point, with the highest increase occurring in the lung tissues of CR3 siRNA group at 24 h. Taken together, these results indicate that the up regulation of CR3 plays a role in the enhanced clearance of *S. aureus* and D39 in PepO-treated mice.

## Discussion

Our previous study has detected the down regulation of SHIP1 and the up regulation of CR3 in PepO-stimulated macrophages. However, the relationship between their changed expression and the enhanced phagocytic function of PepO-stimulated macrophages has not been proven. In this study, we provide evidence that PepO enhances the phagocytic function of macrophages in a SHIP1 and CR3 dependent manner. The down regulation of SHIP1 mediates the up regulation of CR3 in PepO-stimulated macrophages. Furthermore, PepO promotes the clearance of *S. aureus* and D39 from nasopharynx and lungs, and the down regulation of SHIP1 and the up regulation of CR3 also play a role in this process.

At least to our knowledge, in this study we first proved the regulation of CR3 expression by SHIP1. The regulation of CR3 activity by SHIP1 has long been investigated in previous studies (Dianne et al., 2001; Horan et al., 2007), while less attention has been given to the regulation of CR3 expression. This study provides new evidence that the expression of CR3 is also regulated by SHIP1. Unexpectedly, we found that both knockdown and over-expression of SHIP1 lead to the up regulated expressions of CR3 transcripts and protein in macrophages without PepO stimulation. The possible reason may be that the regulation of CR3 expression by SHIP1 is dynamic and needs to be balanced. SHIP1 probably not merely inhibits CR3 expression. Under a certain circumstance, it may also promote CR3 expression. Numerous studies have demonstrated that SHIP1 is a multifunctional protein controlled by various regulatory inputs and regulates downstream signaling via multiple means (Pauls and Marshall, 2017). Both activation and inhibition of SHIP1 inhibit the phosphoinositide 3-kinase signaling pathway (Ong et al., 2007; Fuhler et al., 2012; Fernandes et al., 2013). Another possibility for this unexpected finding is that SHIP1 over-expression activates other signaling molecules, which may mediate the up regulation of CR3. Based on the complexity of immune cell signaling regulation by SHIP1, we speculate that the regulation of CR3 expression by SHIP1 should be a complicated process and to understand this process, further investigation is still needed.

*Streptococcus pneumoniae* and *S. aureus* are dominant colonization bacteria in the upper respiratory tract (Zemlickova et al., 2006; Thapa et al., 2017; Dunne et al., 2018; Lo et al., 2019). Both *S. aureus* strain and *S. pneumoniae* strain used in our study were strong pathogenic strains. PepO promotes the clearance of these strains from nasopharynx and lungs, confirming its protective effect as an immunomodulation agent. The protective effects of gastrointestinal microorganism and their metabolites against infections have long been investigated in previous studies (Ubeda et al., 2013; van Nood et al., 2013; Singh et al., 2014; Buffie et al., 2015; Caballero et al., 2015; Stripling et al., 2015; Lewis and Pamer, 2017; Deng et al., 2019), while less attention has been given to the respiratory microorganisms, especially respiratory pathogens. At least to our knowledge, this study proved for the first time that product from respiratory pathogens can also play protective roles against respiratory infections.

Our current study demonstrated the effectiveness of PepO against specific strains of *S. aureus* and *S. pneumoniae*, and whether PepO can promote the clearance of additional strains of these species including antibiotic resistant isolates (e.g., methicillin-resistant *Staphylococcus aureus* (MRSA) or antibiotic resistant *S. pneumoniae*) and additional species including gram-negative bacteria (e.g., *Pseudomonas aeruginosa* or *E. coli*) still needs to be further determined. Theoretically speaking, PepO can also promote the clearance of these organisms, because it plays this role via enhancing the phagocytosis by macrophages. Even so, further experiment data are still needed to support the hypothesis.

PepO is a multifunctional pneumococcal virulence protein. Agarwal et al., demonstrated that it facilitates host cell invasion and evasion of innate immunity through interaction with plasminogen, fibronectin, C1q, and C4BP (Agarwal et al., 2013, 2014). Our work proved that PepO promotes innate immune activation via TLR2 and TLR4 signaling pathways (Zhang et al., 2016; Yao et al., 2017; Shu et al., 2020). These conclusions seem to contradict each other. Agarwal et al., concentrated on the role of PepO as a virulence protein during pneumococcal infection, and we focused on its role as a pathogen associated molecular pattern without infection, which constitutes the dual character of virulence factor. The innate immune activation effect lays the basis for PepO as an anti-microbial agent, and this effect is associated with TLR2/4 activation, which indicates that protein with similar function may be a new source of antimicrobial agents.

In summary, this study demonstrates that PepO promotes the clearance of *S. aureus* and *S. pneumoniae* from nasopharynx and lungs, and the down regulation of SHIP1 and the up regulation of CR3 play a role in this process. Our study provides a new preventive and therapeutic option for respiratory infectious diseases and lays the theoretical basis for the development of PepO as an immunomodulation agent.

## Data Availability Statement

The original contributions presented in the study are included in the article/supplementary material, further inquiries can be directed to the corresponding author/s.

## Ethics Statement

This animal study was reviewed and approved by the Ethics Committee of Chongqing Medical University.

## Author Contributions

HZ, YY, and XZ planned the experiments. SL, JX, YM, and ZS performed the experiments. HZ, TY, WX, and XZ analyzed the data. HZ and XZ wrote the paper. All authors contributed to the article and approved the submitted version.

## Conflict of Interest

The authors declare that the research was conducted in the absence of any commercial or financial relationships that could be construed as a potential conflict of interest.
